# Novel Immune-Based treatments for Diffuse Large B-Cell Lymphoma: The Post-CAR T Cell Era

**DOI:** 10.3389/fimmu.2022.901365

**Published:** 2022-06-01

**Authors:** Suheil Albert Atallah-Yunes, Michael J. Robertson, Utpal P. Davé, Paola Ghione, Fabiana Perna

**Affiliations:** ^1^Department of Medicine, Division of Hematology/Oncology, Indiana University School of Medicine, Indianapolis, IN, United States; ^2^Department of Medicine, Division of Hematology/Oncology Richard L. Roudebush VA Medical Center, Indianapolis, IN, United States; ^3^Lymphoma and Myeloma Program, Roswell Park Comprehensive Cancer Center, Buffalo, NY, United States

**Keywords:** diffuse large B-cell lymphoma, immunotherapy, chimeric antigen receptor T-cell therapy, target, discovery, high-risk

## Abstract

Prognosis for patients with refractory/relapsed (R/R) diffuse large B-cell lymphoma (DLBCL) is poor. Immune-based therapeutic treatments such as CD19 Chimeric Antigen Receptor (CAR) T cell therapies have dramatically changed the treatment landscape for R/R DLBCL leading to durable remissions in ~ 50% of patients. However, there remains an unmet need for developing novel therapies to improve clinical outcomes of patients not responding or relapsing after CAR T cell therapies. Lack of suitable immunotherapeutic targets and disease heterogeneity represent the foremost challenges in this emerging field. In this review, we discuss the recently approved and emerging novel immunotherapies for patients with R/R DLBCL in the post-CAR T era and the cell surface targets currently used.

## Introduction

Diffuse large B-cell lymphoma (DLBCL) is the most common type of non-Hodgkin lymphoma (NHL). It comprises about 30-40% of all NHL cases in the United States (1–3). The R-CHOP regimen (Cyclophosphamide, Doxorubicin, Vincristine, Prednisone combined with Rituximab, a monoclonal antibody targeting CD20), represents the standard of care for upfront treatment of DLBCL leading to cure in about 60 to 65% of cases (4).

Subtypes of DLBCL associated with poor response include the activated B-cell (ABC)-like subtype (5–7), double expressor lymphomas (DELs) and double/triple hit lymphoma (DHL/THL) (8). DELs comprise 50% of refractory/relapsed (R/R) DLBCL cases and are defined by overexpression of *MYC* and *BCL-2*. DHLs/THLs comprise 6 to 14% of DLBCL cases and refer to the presence of rearrangement in the *MYC* gene in addition to *BCL2* or/and *BCL6* genes. Meta-analysis and retrospective studies initially showed a superior progression-free survival (PFS) with upfront intensification treatment in patients with DHL/THL which led to the common practice of using upfront DA-EPOCH (dose-adjusted etoposide, prednisone, vincristine, cyclophosphamide, doxorubicin) instead of R-CHOP in this patient subset (9, 10). However, DA-EPOCH is also associated with higher toxicity with no evidence of improving overall survival (OS). After induction with DA-EPOCH, consolidation therapy with autologous stem cell transplantation (ASCT) in patients in first remission, did not show improved outcomes compared to R-CHOP (1, 11).

Patients who present with R/R DLBCL have poor prognosis with overall response rate (ORR) and complete remission (CR) of 26% and 7% respectively and a median OS of 6 months (12). High-dose chemotherapy followed by ASCT has been a standard treatment for R/R DLBCL patients with chemo-sensitive disease after salvage therapy. However, durable remissions after consolidative ASCT occur in only about 50% of patients (13, 14) and outcomes with ASCT are worse in patients bearing the aggressive subtypes mentioned above (15). In a retrospective analysis of 177 R/R DLBCL patients who underwent ASCT after showing chemosensitivity, 4-year PFS and OS were 28% and 25% respectively in those who had DHL compared to 57% and 61% respectively in patients without DHL. Those who had DEL had 48% 4-year PFS (16).

Patients who are not cured with ASCT or are ineligible for ASCT due to age and/or comorbidities or have chemo-refractory disease after salvage chemotherapy, may be considered for Chimeric Antigen Receptor (CAR) T cell therapy targeting CD19. CARs are recombinant receptors that can redirect the specificity and function of cytotoxic T-lymphocytes to target antigen such as CD19. The CAR T cells act as “living drugs” that exert both immediate and long-term effects. The engineering of CARs requires the *in vitro* culture, transduction, and expansion of primary autologous T cells. Stable gene transfer is required to enable sustained CAR expression in clonally expanding and persisting T cells. In principle, any cell surface molecule can be targeted through a CAR, thus over-riding tolerance to self-antigens and the antigen recognition gaps in the physiological T-cell repertoire that limit the scope of T-cell reactivity (17).

Three autologous CD19 CAR T cell products: axicabtagene ciloleucel (axi-cel), tisagenlecleucel (tisa-cel) and lisocabtagene maraleucel (liso-cel) have been approved by the Food and Drug Administration (FDA) for the treatment of R/R DLBCL after two prior lines of therapy (18–22). Responses seemed to occur regardless of the cell of origin (ABC vs GC subtypes) or DHL/THL status. Despite the high rate of complete responses seen with CAR T cell therapy, only 30%-40% of patients achieve durable remissions (3).

Most recent results from the ZUMA7 (NCT03391466) (23) and TRANSFORM (NCT03575351) trials showed that in R/R DLBCL patients within 12 months from the first line of therapy, receiving CAR T cell therapy improved event free survival (EFS) and response rates compared to R/R DLBCL patients who received standard of care (salvage chemotherapy and ASCT). At a median follow up of 24.9 months, median EFS in the ZUMA7 trial was 8.3 months for the axicabtagene ciloleucel arm vs 2 months in patients who received standard of care. In the interim analysis of the TRANSFORM trial, median EFS in the 92 patients who received lisocabtagene maraleucel was 10.1 compared to 2.3 months among the 92 patients treated with standard of care. These data were contrasted by the BELINDA (24) study (NCT03570892), using tisagenlecleucel, which showed no difference in outcomes.

Allogeneic stem cell transplant (allo-SCT) is a potential option for patients with R/R DLBCL but is mainly reserved for medically fit patients with disease progression after ASCT and/or CAR T cell therapy. A retrospective analysis reported 50-60% long term survival after allo-SCT but this therapeutic modality has a 40-50% treatment-related mortality (25). Retrospective analysis of a small sample of patients with DHL/THL who underwent allo-SCT showed similar outcomes (PFS, OS) to those who did not have DHL/THL (26).

Given that, there is an urgent need for novel therapeutic approaches in high-risk R/R DLBCL.

While multiple targets are currently evaluated in clinical trials for treatment of DLBCL such as NF-κB and PI3K/AKT/mTORC pathways, Bruton’s tyrosine kinase (BTK), Spleen tyrosine kinase (SYK), enhancer of zeste hormone 2 (EZH2), phosphoinositide-3-kinase (PI3K), Janus Kinases (JAK), and anti-apoptotic proteins (e.g. BCL2, MCL1) (27, 28), we will mainly focus on immune-based therapeutic interventions. Immunotherapy is a promising cancer treatment that relies on the identification of suitable cell surface molecules (29).

## Immune-Therapeutic Approaches Targeting CD20 and CD19

The clinical success of rituximab and CAR T cell therapy in DLBCL paved the way to several immunotherapeutic drugs for the treatment of R/R DLBCL. Based on their mechanisms of action they can be divided into 1) monoclonal antibodies (mAbs), 2) antibody drug conjugates (ADC), 3) bispecific T-cell engagers (BiTE). MAbs result in direct toxicity upon binding to a target surface antigen and promote antibody-dependent cellular cytotoxicity (ADCC), antibody-dependent cellular phagocytosis (ADCP), and complement-mediated killing of tumor cells (30, 31). ADCs are comprised of mAbs linked to cytotoxic drugs that are internalized into the tumor cell after binding of the mAb to a specific surface antigen (32). Bi-specific antibodies also recognize target antigens on immune cells such as CD3 on T-cells in addition to lymphoma cells (33).

### Monoclonal Antibodies

In the pre-rituximab era, CHOP chemotherapy alone was the standard treatment for DLBCL curing less than 50% of patients (30, 34). Rituximab received FDA-approval in 2006 as frontline treatment of DLBCL in combination with CHOP or CHOP-like based therapies and has improved clinical outcomes of patients with DLBCL (35–37). More recent mAbs that target CD20 include ofatumumab and obinutuzumab. Ofatumumab is a mAb that binds to a different CD20 epitope than the one recognized by rituximab and results in a more potent complement-dependent cytotoxicity than rituximab. Obinutuzumab is a mAb with a modified Fc portion which provides a more potent ADCC activity than rituximab and less potent complement-dependent cytotoxicity (38). In a randomized phase 3 study of obinutuzumab-CHOP versus rituximab-CHOP, obinutuzumab did not show superior outcomes in DLBCL patients compared to rituximab but resulted in more adverse events including infections, fever, and cytopenias (39–41). Radiolabeled CD20 mAbs such as ibritumomab tiuxetan (Zevalin) and I-131 tositumomab (Bexxar) are also used in treatment of DLBCL (42, 43).

Tafasitamab recognizes CD19 and results in more potent ADCC and ADCP activities than rituximab against lymphoma cells (44, 45). Tafasitamab was developed with the overall goal of overcoming resistance to rituximab especially after multiple rituximab-based therapies (44, 45). In a phase 2 study of 92 R/R NHL patients ineligible for ASCT including 35 R/R DLBCL patients with 74% of them maintaining a clinical response for less than 12 months, tafasitamab monotherapy resulted in a 26% ORR (6% CR). At long-term follow-up (≤4 years), median PFS was 2.7 months and 12-months rate PFS was 34% for the R/R DLBCL subgroup. Median duration of response (DOR) was 20.1 months (46, 47). Patients with a baseline number of peripheral blood NK cells greater than 100 cells/µL had better PFS (8.8 months vs 2.3 months) (46). Combining lenalidomide, an immunomodulatory drug, to tafasitamab enhanced NK cell activity and ADCC against lymphoma cells (45, 48). At data cut off (October 2020), the phase 2 L-MIND trial of tafasitamab + lenalidomide given to 81 patients with R/R DLBCL ineligible for ASCT and receiving a maximum of 3 previous lines of treatment reported 57.5% ORR including 40.0% CR. PFS was 11.6 months at a median follow up of 33.9 months and median OS was 33.5 months in a median follow up of 42.7 months. Median DOR was 43.9 months and not reached in those who achieved CR (49, 50). The two patients with DHL/THL and seven out of eight patients with transformed lymphoma responded as well. This led to the accelerated approval of tafasitamab in combination with lenalidomide by the FDA in August 2020 for treatment of R/R DLBCL patients ineligible for ASCT. The OS and PFS were superior in patients who received only 1 prior line of treatment than those who received 2 or more lines (50).

It is unclear if tafasitamab could impair CD19 CAR T cell activity (51). Similarly, whether it is better to consider tafasitamab in R/R DLBCL prior to CAR T cell therapy or after disease progression remains unknown and most practices take patients to CAR T cell therapy first because of the higher sensitivity of CAR T cells compared to mAbs. Responses in patients with ABC subtypes in the L-MIND study were favorable (50). Other ongoing studies of tafasitamab in the R/R DLBCL setting include the combination of tafasitamab plus bendamustine and rituximab (BR) vs BR alone.

### Antibody-Drug Conjugates

An ADC is an antibody linked to a cytotoxic agent (payload). After the antibody binds to the target antigen on tumor cells, the complex gets internalized into the cell and the linker gets degraded releasing the cytotoxic agent (32).

Loncastuximab tesirine is an ADC comprising a humanized CD19 mAb conjugated to a pyrrolobenzodiazepine dimer toxin *via* a protease-cleavable valine–alanine linker. It is approved as a third line treatment in patients with R/R DLBCL. Approval was based on the updated analysis of data from the phase 2 LOTIS-2 trial in which 145 patients with R/R DLBCL had 48% ORR, 24% CR and a median DOR of 10.3 months after a median follow up of 7.3 months. ORR in patients with DHL or THL was 33% (all CR). In those who failed CAR T cell therapy, ORR was 46% (52). The most common adverse side effects reported with locastuximab tesirine involve hematologic toxicities followed by liver toxicity, nausea and edema (52, 53).

Coltuximab ravtansine is another ADC targeting CD19. It is a complex of CD19 mAb linked to DM4 (tubulin toxin derived from maytansine) *via* N-succinimidyl-4-(2-pyridyldithio)butyrate glutathione-sensitive linker. Coltuximab ravtansine led to an ORR and CR of 43.9% and 14.6% respectively in the phase 2 study of 41 patients with R/R DLBCL (54).

MT3724 is a novel CD20 directed ADC linked to a ribosome inhibitor derived from shiga-like toxin that was tested in a phase 1 study of R/R DLBCL leading to 30% ORR (42).

### Bispecific Antibodies

Bispecific engagers (BiE) are antibodies with specificities for two distinct surface antigens allowing effector immune cells such as T-cells, NK-cells and macrophages to be brought into proximity to NHL cells. This enhances the cytotoxic activity of the effector cells against the cancer cell. The structure of bispecific antibodies may lead to various forms with different pharmacokinetics and pharmacodynamics. For example, Fc-free BiEs are rapidly cleared from the body due to their small molecular size and may require administration as a continuous intravenous infusion. However, removing the Fc portion may allow more penetration to tumor tissue. IgG like BiEs have full or modified Fc regions to bind more specifically to T-cells and less to other immune effector cells such as macrophages and NK cells (33, 55). Early phase studies showed that bispecific antibodies could induce CR in patients with R/R DLBCL including those who received previous CAR T cell therapy (56–58).

Most of the Bispecific T-cell Engagers (BiTEs) under development for treatment of DLBCL engage the CD3 invariant subunit of the T-cell receptor complex and CD19 (CD19 x CD3 BiTE) or CD20 (CD20 xCD3 BiTE) on lymphoma cells (33, 55).

Blinatumomab is the first FDA-approved CD19xCD3 BiTE for clinical use as a second line treatment of B-cell acute lymphoblastic leukemia (B-ALL). Early clinical trials showed promising results with blinatumomab in patients with R/R B-cell NHL. In the phase 1 trial by Goebeler et al, blinatumomab resulted in ORR of 69% (37% CR + CRu and 31% PR) in 35 patients with NHL (2). ORR in the subset of patients who had DLBCL was 55% (59). Blinatumomab was tested in a phase 2 study that included 21 patients with heavily treated R/R DLBCL. ORR and CR were 43% and 19% respectively (60). Most common reported side effects included tremors, pyrexia, edema, and fatigue. Aphasia or encephalopathy occurred in 9%. Somnolence, disorientation, dizziness, or speech problems occurred in 4%. 4/5 patients who discontinued the stepwise dosing had neurologic adverse events that eventually resolved (60). In the phase 2 study of stepwise blinatumomab in 41 patients with R/R aggressive NHL (68% refractory) patients did not achieve CR after salvage chemotherapy, ORR and CR after 12 weeks were 37% and 22% respectively. Enrolled patients had GCB and non-GCB subtypes and 9 patients had DHL or THL. 8 responders (CR or PR) were able to receive stem cell transplant subsequently indicating that blinatumomab could bridge R/R DLBCL patients to auto/allo SCT, especially those who fail to achieve good responses to first salvage regimens (61). A phase 2 study of blinatumomab was conducted in patients achieving CR/PR/SD with R-CHOP in new high risk DLBCL (High international prognostic index (IPI) and DHL/THL patients) (62). ORR after 12 weeks of starting blinatumomab was 89% (25/28 patients). The 4 patients who did not achieve metabolic response with R-CHOP responded to blinatumomab. The common CRS and neurotoxicity with blinatumomab and the continuous intravenous administration of blinatumomab due to its short half-life makes it inconvenient for some patients. Reversible high-grade CRS and neurotoxicity occurred up to 22% of patients in the blinatumomab phase 1 trial. Hence dexamethasone is administered prior to the first dose and with every dose escalation (33, 63). Early studies with the strategy of combining blinatumomab with other immunotherapeutic agents are ongoing (33, 63). Blinatumumab is being tested in combination with pembrolizumab in a phase 1b study in patients with R/R DLBCL (NCT03340766).

Mosunetuzumab is a fully length humanized IgG bispecific antibody. It is the first in-human CD20 X CD3 BiTE. In the phase 1 study by Budde et al., including patients with heavily treated R/R DLBCL, mosentuzumab was given in 3 week cycles (56). At data cutoff (January 2020), analysis of the 129 patients from group B with R/R aggressive NHL reported ORR and CR of 34.9% and 19.4%, respectively. Median DOR for the aggressive NHL subset was 7.6 months and increased to 22.8 months in those who achieved CR. Adverse events were confined to cycle 1 and occurred in ≥ 20% of patients: neutropenia (28.4%), CRS (27.4%) and mostly low grade, hypophosphatemia (23.4%), fatigue (22.8%), and diarrhea (21.8%). It was concluded that stepwise dosing of mosunetuzumab is safe with manageable toxicity and can lead to durable CR in patients with R/R DLBCL (56). It is unknown whether mosunetuzumab is effective in patients relapsing after CAR T cell therapy. 19 patients in the phase 1 trial by Budde et al. relapsed after previous CAR T cell therapy and included 14 patients with aggressive NHL. After receiving mosenutuzumab, the ORR and CR in this subset of 19 patients were 36.8% and 26.3%, respectively. This indicates that mosunetuzumab may be a potential option in R/R DLBCL patients after receiving prior CAR T cell therapy. Ongoing studies in R/R DLBCL evaluate the combination of mosunetuzumab with other therapeutics including polatuzumab based regimens (57), gemcitabine-oxaliplatin (NCT04313608), and immune checkpoint inhibitors (NCT02500407). Also, mosunetuzumab will be evaluated in combination with R-CHOP or R-CHOP + polatuzumab at the frontline treatment of DLBCL (NCT03677141).

Glofitamab is another CD20 xCD3 BiTE that has one binding site for CD3 on the T-cell and two binding sites for CD20 on the neoplastic B cells (64). In the phase 1 study by Hutchings et al. involving 171 patients with R/R NHL including 127 patients with heavily pretreated (90.6% refractory) aggressive NHL (R/R DLBCL, transformed forms) (58). In the R/R aggressive NHL subgroup with stepwise dosing, ORR and CR were 61% and 54% respectively. In this study, obinutuzumab was given 7 days prior to cycle 1 of glofitamab to attempt decreasing the B-cell lymphoma burden and mitigating CRS. It was concluded that glofitamab can lead to meaningful and durable responses in heavily pretreated R/R DLBCL regardless of disease burden. Only 3 patients in the study received prior CAR T cell therapy thus conclusions cannot be made in regards to this subset of patients. Adverse events and CRS with glofitamab were mostly confined to cycle 1 and were low grade and self limited (58). Ongoing and recruiting trials will evaluate the role of glofitamab with gemcitabine-oxaliplatin (NCT04313608), polatuzumab (NCT03533283), immune check point inhibitors (NCT03533283) in the R/R DLBCL setting. Also, glofitamab will be tested after prior CAR T cell therapy (NCT04889716) and in combination with standard chemotherapy in newly diagnosed DLBCL (NCT04914741).

Plomatamab is another humanized CD20 xCD3 BiTE that is modified for better potency and safety. Plomatamab was evaluated in a stepwise dose a phase one trial of R/R NHL. 13 (38.2%) of the patients with R/R DLBCL responded including 28% with CR. At a high dose of 170 μg/kg, ORR and CR increased to 50% and 25% respectively. 4 of the 16 patients who received prior CAR T cell therapy also responded (65).

Epcoritamab is a CD20 xCD3 BiTE that showed promising results in the phase 1/2 EPCOR trial by Hutchinson et el. 73 patients with R/R NHL (68% DLBCL) were enrolled to receive escalating doses of epcoritamab. 68 patients received the drug. The ORR and CR in the heavily pretreated R/R DLBCL subgroup at full doses of 12-60 mg were 68% and 45% respectively. ORR and CR were 88% and 38% in those who received a dose of 48 mg which was the dose recommended for the phase 2 study. All 4 patients with R/R DLBCL who received prior CAR T cell therapy responded with 2 achieving CR. At a dose of 48 mg, CRS occurred in about 67% of patients however was grade 1 or 2 (66, 67). A phase 3 trial of epcoritamab vs standard of care will be conducted in R/R DLBCL patients (68). The addition of epcoritamab to frontline R-CHOP is also being evaluated in newly diagnosed high-risk DLBCL patients with preliminary results for a small number of patients indicating its safety and a manageable toxicity profile (69).

Odronextamab is another fully human IgG4 CD20 X CD3 BiTE with a modified Fc domain that is being studied in lymphoma. In an escalating dose phase 1 study of intravenous odronextamab including 71 patients with R/R DLBCL, ORR was 60% (all CR) with a median DOR of 10.3 months in those who did not receive prior CAR T cell therapy and received doses ≥80 mg of odronextamab. Those patients who received prior CAR T cell therapy had ORR and CR of 33.3%, and 23.8% respectively with a median DOR of 2.8 months. Most of the CRs, regardless of prior CAR T cell therapy, are ongoing. Toxicity was manageable and similar to what was seen with other BiTEs (70).

The most concerning side effects of BiTE are CRS and neurotoxicity due to overactivation of T cells including those within the CNS. Attempts to mitigate the risk and severity of CRS include subcutaneous administration of the drug, stepwise dosing, and pretreatment with other regimens to decrease the B-cell burden. Lymphopenia is common as CD19 and CD20 antigens are also expressed on normal B cells but this can be compensated by administration of IVIG until restoration of the B-cell lineage (33, 55).

## Immune-Therapeutic Approaches Targeting Molecules Other Than CD20 and CD19

Loss of CD19 expression in some patients exposed to CD19 CAR T cell therapy represents a mechanism of resistance and relapse and it is generally defined as “antigen escape” (71–73). In the phase 1 trial of a CD19-4-1BB-ζ CAR for pediatric B-ALL, 13 of 55 patients (24%) had a CD19-negative relapse after achieving a prior CR (72). In the global phase 2 trial of tisagenlecleucel for B-ALL in children and young adults, 15 of 16 patients who relapsed had CD19-negative disease (71). In NHL, the role of antigen escape after CD19 targeted CAR T cell therapy is less well defined (74). In the combined phase 1 and 2 trials of axicabtagene ciloleucel in NHL, 3 out of the 11 patients who relapsed had tissue available for analysis, and had CD19-negative disease (75). In a trial of CD19 targeted CAR T cell therapy in NHL, 5 non-responders were found to have CD19-negative disease (76). Similarly, the loss of CD20 has been reported following rituximab therapy in lymphoma patients (77). Thus, there is an increasing interest in finding novel targets other than CD19 and CD20 for treatment of R/R DLBCL patients. **Table 1** shows targeted cell surface molecules in NHL.

**Table 1:** Targeted cell surface molecules in treatment of NHL.

Cell surface targets	Biological function	Targeted therapies	FDA approval status and indications in R/R DLBCL
CD19	Co-receptor for the B-cell antigen receptor complex (BCR). It decreases the threshold for activation of downstream signaling pathways.	Tafasitamab (Mab) Loncastuximab tesirine, Coltuximab ravtansine (ADC) Axicabtagene ciloleucel, Tisagenlecleucel, Lisocabtagene maraleucel (CAR)	Tafasitamab in combination with lenalidomide is approved for treatment of R/R DLBCL patients ineligible for ASCT. Approval was based on the phase 2 L-MIND trial (NCT02399085) Loncastuximab tesirine is approved as a third line treatment in patients with R/R DLBCL based on the phase 2 trial (NCT03589469). Axicabtagene ciloleucel, Tisagenlecleucel, Lisocabtagene maraleucel (CAR) are FDA approved for the treatment of R/R DLBCL after two prior lines of therapy Coltuximab ravtansine is not approved. It was tested in a phase 2 trial in patients with R/R DLBCL
CD20	It promotes development, differentiation, and activation of B-lymphocytes.	Rituximab, Obinutuzumab, Ofatumumab (MAb) Ibritumomab tiuxetan, Iodine-131 tositumomab (radiolabeled mab) MT3724 (Engineered toxin body)	Rituximab approved for treatment of R/R DLBCL in various settings. Iodine 131-tositumomab is approved for treatment of R/R transformed follicular lymphoma. MT3724 was tested in phase 1 trial. Phase 2 study is ongoing (NCT02361346)
CD19 X CD3		Blinatumumab (BiTE)	Not FDA approved. Blinatumomab was tested in phase 2 trials in R/R DLBCL (NCT01741792). Most phase 3 trials in R/R DLBCL were held due to modest activity and presence of more promising immunotherapeutic agents
CD20 X CD3		Mosenutuzumab, Glofitamab, Plomatamab, Epcoritamab, Odonextamab(BiTE)	-Not FDA approved -BiTEs were tested in phase 1 trials in R/R DLBCL with promising results (NCT03075696) (NCT02500407) (NCT03671018) (NCT03625037) (NCT02290951) (NCT02924402) -Phase 2 trials are ongoing. -Phase 3 trials are ongoing (NCT04628494) (NCT04408638) (NCT05171647)
CD22	It mediates B-cell interactions and localization in lymphoid tissues. It regulates BCR signaling.	Epratuzumab (mAb) Pinatuzumab vedotin, Inotuzumab ozogamycin (ADC)	-Not FDA approved -Epratuzumab has been tested in a phase 2 trial including patients with R/R DLBCL. Pinatuzumab vedotin (NCT01691898) and inotuzumab ozogamycin (NCT00867087) have been tested in phase 2 trials. Phase 3 trial of inotuzumab was discontinued.
CD30	It activates NF-κB transcription	Brentuximab vedotin (ADC)	-Not FDA approved for R/R DLBCL but used occasionally off label when CD30 is expressed and especially in patients unfit for chemotherapy. -Brentuximab + lenalidomide + rituximab regimen is being compared against placebo + rituximab + lenalidomide in a phase 3 trial (ECHELON-3) of R/R DLBCL patients
CD79b	Along with CD79a, it initiates the signal transduction cascade activated by BCR which leads to internalization of the complex, trafficking to late endosomes and antigen presentation.	polatuzumab vedotin (ADC)	Polatuzumab vedotin was approved in combination with bendamustine + rituximab for treatment of R/R DLBCL patients who are ineligible for ASCT and after two lines of therapy. Approval came based on initial results of the phase 2 trial (NCT02257567)
CD47	Macrophage checkpoint that provides “do not eat me signal”	magrolimab (mab)	Not FDA approved. Studied in a phase 1 and 2 (NCT02953509) in patients with R/R DLBCL. Phase 2 studies (NCT03309878) are ongoing

### CD30

Brentuximab vedotin is a compound of CD30 mAb linked to cytotoxic moiety monomethyl auristatin (MMAE) which disrupts the microtubules leading to apoptosis of mainly proliferating lymphoid neoplastic cells. MMAE is linked to the CD30 mAb *via* a protease-sensitive dipeptide valine–citrulline linker that is cleaved after internalization into the cell to release MMAE (78). CD30 gets rapidly internalized after binding to mAb. Brentuximab vedotin may also have a bystanding effect on neighboring tumor cells that do not express the surface target possibly by traveling of the cytotoxic moiety through cell membranes to other cells explaining the responses seen with brentuximab vedotin in patients with CD30 expression of <10%. Brentuximab vedotin is approved for use in treatment of newly diagnosed advanced stage classical Hodgkin lymphoma, R/R classical Hodgkin lymphoma (HL) and as a consolidative treatment after ASCT in high-risk classical Hodgkin lymphoma patients. However, up to 25% of DLBCL cases express CD30. In DLBCL, its use as monotherapy is mostly limited to patients who have CD30 expression in the R/R setting or in patients who are unfit for chemotherapy (78).

AFM13 is a bispecific NK cell engager that targets CD16A on NK cells and CD30 on NHL and Hodgkin lymphoma tumor cells. To increase activation of the NK cell and prevent the degradation of CD16A by metalloproteinases, several strategies are being implemented including the addition of metalloproteinase inhibitors, targeting more than one receptor on the NK-cell, and incorporating IL-15 making trispecific NK cell engagers (33).

### CD79b

Polatuzumab vedotin is an anti-CD79b mAb linked to MMAE *via* a protease-sensitive dipeptide valine–citrulline linker. CD79a and CD79b are part of the heterodimeric signaling component of the B-cell receptor. After binding to antigens or polatuzumab vedotin, the BCR complex gets internalized into a lysosomal-like endocytic component which provides a suitable environment for cleavage of the linker in polatuzumab vedotin. Such property along with the expression of CD79 almost limited to B-lymphocytes paved the way for introducing polatuzumab vedotin in the treatement of lymphoid malignancies (32, 45). Polatuzumab vedotin led to positive outcomes in R/R DLBCL patients when combined with mAbs including rituximab and obinutuzumab (32, 79).. Polatuzumab in combination with BR is a recently approved regimen for treatment of R/R DLBCL, specifically in patients who are ineligible for ASCT and after two lines of therapy. The phase 2 trial of BR alone versus polatuzumab plus BR in 80 patients with R/R DLBCL resulted in ORR and CR of 45% and 40% respectively in the group that received polatuzumab and 17.5% of each ORR and CR in the BR without polatuzumumab study arm. Median PFS, OS and DOR were 7.6 months, 12.4 months and 12.6 months respectively in the BR + polatuzumab group compared to 2.0 months,4.7 months and 7.7 months, respectively in the BR alone study arm. Responses were seen regardless of cell of origin subtype, degree of CD79b expression, and MYC/BCL2 double expression (80).

In practice, polatuzumab plus BR may be given as a bridge to CAR T cell therapy or allo-SCT. In a retrospective analysis of 80 patients with DLBCL, 7 of 12 patients who received polatuzumab after CAR T cell therapy as a palliative therapy or as a bridge to allo-SCT, responded. This indicates that polatuzumab is likely active in R/R DLBCL patients who failed prior CAR T cell therapy (81). In a multicenter retrospective study that looked into 57 patients with R/R DLBCL who were R/R to CAR T cell therapy and received polatuzumab with or without bendamustine and rituximab after CAR T cells, ORR and CR were 44% and 14% respectively. After a median follow up of 47 weeks, median PFS was 10 weeks and 80% of patients progressed or died with subanalysis showing a trend of shorter PFS in patients with bone marrow involvement and LDH elevations indicating that durability of responses to polatuzumab after CAR T cell therapy may be short in patients with high tumor burden (82). Several ongoing early phase trials in the R/R DLBCL setting are studying the combination of polatuzumab with other therapeutic targets, including venetoclax, lenalidomide, and bispecific antibodies (32).

The results for the POLARIX phase 3 trial in newly diagnosed DLBCL patients with IPI scores of 2-5 were recently published (83). Enrolled subjects received either 6 cycles of R-CHOP (N=439) or 6 cycles of polatuzumab + R-CHP (N=440), both groups received additional 2 cycles of rituximab after the 6 cycles of chemotherapy; 40% of patients had double expressor DLBCL and 7% had double or triple hit DLBCL. At 2 years, PFS for the polatuzumab- based regimen was 76.7% compared to 70.2% in the R-CHOP group. Although OS and responses were similar in the two groups, the patients who progressed on R-CHOP group required more subsequent therapy. The safety profiles between the 2 randomized arms were similar. Upon further analysis, most PFS benefit was seen patients older than 60, those who have ABC histologic subtype, and high IPI scores of 3-5 (83, 84). The POLARIX study showed that polatuzumab + CHOP may be the first regimen to be incorporated in frontline DLBCL after several studies failed to improve outcomes of R-CHOP within the last two decades. Main side effects of polatuzumab plus BR include peripheral neuropathy and cytopenias. Neuropathy is dose and duration dependent, it is recommended to hold polatuzumab in those who develop high grade neuropathy until improvement to grade 1 or total resolution with subsequent dose reduction (32).

### CD22

Epratuzumab, an anti-CD22 mAb was tested in 6 R/R DLBCL with 67% and 50% ORR and CR rate respectively (85). Pinatuzumab vedotin is an anti-CD22 ADC with the payload being MMAE. When it was combined with rituximab in a group of R/R DLBCL, ORR and CR were 60% and 26%, respectively (86).

## Immune Checkpoint Inhibitors

Immune checkpoint inhibitors are drugs that block signaling pathways that attenuate immune cell activation, thereby enhancing the cytotoxic activity against cancer cells (87). CTLA-4 and PD-L1/PD-1 ligand/receptor pair are the most common checkpoints targeted by mAbs (87). Despite the success of immune checkpoint inhibitors in treating solid tumors, inhibition of the PD-1/PD-L1 axis has led to fewer responses in R/R DLBCL (88, 89). This may be due to the lower mutational burden of hematologic malignancies compared to solid tumors and thus, the reduced presence of neoantigens enabling cancer cell recognition by the immune cells. Thus, PD-1/PD-L1 inhibitors are mostly studied in combination with other therapeutics in R/R DLBCL patients. Herrera et al. investigated the combination of ibrutinib + durvalumab in a phase 1b/2 study of patients with R/R FL and R/R DLBCL (GCB DLBCL N=16, non-GCB DLBCL N=16, unspecified DLBCL N=2). In the R/R DLBCL subgroup, the combination led to an ORR of 13% in the GCB subtype and 38% in the non-GCB subtype. In the whole group of R/R FL and DLBCL patients, median PFS was 4.6 months and median OS was 18.1 months, both being shorter in R/R DLBCL. The authors concluded that adding durvalumab to ibrutinib produced similar responses to previous studies with ibrutinib monotherapy however with the addition of immune-related toxicity (90). The frequent genetic aberrations at chromosome 9p24 and overexpression of PD-L1 in primary mediastinal large B-cell lymphoma (PMBCL) makes this lymphoma subtype susceptible to immune checkpoint inhibitors. The results from the phase 2 KEYNOTE-170 trial showed meaningful responses with durable remissions with pembrolizumab monotherapy in patients with R/R PMBCL which led to the FDA approval of pembrolizumab in R/R PMBCL after two or more prior lines of therapy (91). Pembrolizumab monotherapy was also evaluated in 12 patients with R/R DLBCL after prior CD19-directed CAR T cell therapy which resulted in 25% ORR (1 CR, 2 PR) (92). Patients who responded had CAR T cells and non–CAR T cells that are less exhausted compared with those who did not respond. At the frontline setting, pembrolizumab was tested with R-CHOP in 30 patients with DLBCL and resulted in ORR and CR of 90% and 77% respectively with a 2-year PFS of 83% at a median follow up of 25.5 months. Longer PFS was seen in patients with higher degree of PD-L1 expression (93). Several attempts have been made to target other checkpoint inhibitors such as LAG-3, TIGIT, TIM-3, and VISTA (94).

Promising results were seen with magrolimab in various hematologic malignancies. Magrolimab is an IgG4 humanized CD47 mAb that inhibits a macrophage checkpoint on cancer cells and thus inhibits their “do not eat me signal “ and disables macrophage immune evasion. A preclinical study showed that magrolimab may re-sensitize large cell lymphoma cells resistant to rituximab and increased phagocytosis by 80% compared to rituximab alone (95). Pooled data from phase 1b and 2 studies of magrolimab plus rituximab demonstrated ORR and CR of 39% and 20% respectively in 46 patients with R/R DLBC after a median follow up of 12 months (96). The most highlighted side effect of magrolimab is anemia due to hemagglutination as a result of CD47 expression on erythrocytic lineages. Other CD47 mAbs showed activity in preclinical models and are being studied in phase 1 studies (97). TTI-621, a CD47 decoy receptor that target CD47/SIRPα is being evaluated in a clinical trial (NCT02663518).

## Conclusions

CAR T cell therapies have dramatically changed the treatment landscape for R/R DLBCL. Durable remissions have been observed in patients with highly refractory DLBCL after treatment with tisagenlecleucel, axicabtagene ciloleucel, and lisocabtagene maraleucel. These CD19-directed CAR T cell therapeutics are produced using second generation CAR constructs that include CD28 or 4-1BB costimulatory domains. It is to be hoped that CAR T cell therapies based upon later generation CAR constructs may improve outcomes for patients with R/R DLBCL. Use of immune checkpoint inhibitors to augment the antitumor efficacy of CAR T cells is also being actively investigated.

Novel immunotherapeutic approaches before, during, or after CAR T cell therapy require further study. Lack of suitable immunotherapeutic targets, disease heterogeneity, and an immunosuppressive microenvironment challenge the development of novel immune-based therapeutic strategies. In an effort to develop precision approaches, the best target is a cell surface molecule highly expressed on lymphoma cells and absent or minimally expressed on normal tissues. Ideally, the target should play a critical role in cancer survival and progression. The methods to identify such critical target molecules are still in development and only recently advanced Mass-Spectrometry analyses and novel bioinformatic tools are enabling the understanding of the contribution that an altered cancer surface proteome makes to cancer development and progression (29). These technologies will enable the selection of novel targets for use in innovative immunotherapies for patients with high-risk DLBCL and further enhance the efficacy of CAR T cell therapy.

## Author Contributions

All authors contributed to gathering of data, writing, editing, and revising of the manuscript. All authors contributed to the article and approved the submitted version.

## Funding

FP receives research grant support from the Leukemia Research Foundation, Indiana University School of Medicine research grants and Lonza. UD was supported in part by Merit Award I01BX001799 from the U.S. Department of Veterans Affairs, Biomedical Laboratory Research and Development Service. PG received research funds from Kite Pharma and the Lymphoma Research Foundation. The funders were not involved in the study design, collection, analysis, interpretation of data, the writing of this article or the decision to submit it for publication.

## Conflict of Interest

PG participated in advisory boards for Astra Zeneca, Daiichi Sanyo, Secura Bio and Kyowa Hakko Kirin.

The remaining authors declare that the research was conducted in the absence of any commercial or financial relationships that could be construed as a potential conflict of interest.

## Publisher’s Note

All claims expressed in this article are solely those of the authors and do not necessarily represent those of their affiliated organizations, or those of the publisher, the editors and the reviewers. Any product that may be evaluated in this article, or claim that may be made by its manufacturer, is not guaranteed or endorsed by the publisher.
